# Reproductive tract tuberculosis and male infertility

**DOI:** 10.4103/0970-1591.42624

**Published:** 2008

**Authors:** Rajeev Kumar

**Affiliations:** Department of Urology, All India Institute of Medical Sciences, Ansari Nagar, New Delhi - 110 029, India

**Keywords:** Azoospermia, genitourinary tuberculosis, infertility

## Abstract

Infertility is an uncommon manifestation of genitourinary tract tuberculosis. Anatomic obstruction by granulomas or distortion of the normal anatomy by fibrosis surrounding the reproductive tract structures is the commonest cause of infertility. The diagnosis is usually based on a suggestive history along with evidence of granulomatous infection on a tissue sample. The management depends on the site of obstruction and surgery is usually helpful only in cases with discrete ejaculatory duct obstruction. However, most other patients are candidates for *in-vitro* fertilization and have a prognosis similar to that in men with other causes of obstructive azoospermia.

## INTRODUCTION

Tuberculosis of the male reproductive tract can result in infertility. The infection can involve any part of the reproductive tract including the testis, epididymis, vas deferens, seminal vesicles, prostate and the ejaculatory ducts. Infertility usually results from the inflammation and scarring that follow the infection, resulting in distortion of the normal anatomy and causing obstruction. Infertility may be the first presentation of genitourinary tuberculosis and patients may have no recollection of any other symptoms.[1] Involvement of the genital tract usually occurs in the reproductive age group. In a study of 40 men with epididymal tuberculosis, the median age was 32 years.[2] This accounts for its presentation as infertility.

## CLINICAL MANIFESTATION

The site of infection and the resultant scarring determine the manifestation of reproductive tract tuberculosis. In infertile men, determination of the site is critical for deciding the line of management and the potential fertility outcome.

### Epididymis/testis/vas deferens

The epididymis is one of the favored sites for tuberculous infection. In a recent review of 69 patients diagnosed with genital tract tuberculosis, the epididymis was found to be involved in over 78%.[3] In another study on sub-Sahara population, among 28 men with genitourinary tuberculosis, the epididymis was the most frequently infected site (58%).[4]

Unlike other parts of the reproductive tract that are usually infected secondarily due to a retrograde spread of infection from the bladder, the globus minor of the epididymis tends to get infected primarily through a hematogenous spread of the bacilli. The high vascularity of the globus minor may be one of the reasons for this preferential involvement. Chronic epididymitis is, in fact, one of the typical manifestations of genitourinary tuberculosis.[5]

**Figure 1:**
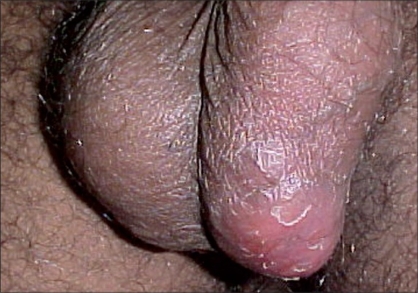
Tubercular scrotal abscess

Tubercular epididymitis may manifest as an acute infection, chronic infection or infertility. Acute inflammation is usually a combined epididymo-orchitis with pain, tenderness and swelling. This may be the commonest manifestation in up to 40% cases.[2] The other common presentation is a scrotal or testicular mass or abscess [Figure 1] with or without pain.[4] Occasionally, the presence of scrotal sinuses adherent to the epididymis may suggest tuberculosis. Bilateral masses may also be present and would usually result in infertility.[6] Infertility may be the presenting feature in about 10% cases and may not resolve following antitubercular therapy.[2] One of the reasons for infertility with epididymal tuberculosis is the frequently bilateral nature of affliction. Chung *et al.*, however, believe that this trend of bilateral involvement in now decreasing.[7]

The testis is a rare site for tuberculous involvement. One of the reasons for this may be the presence of a bloodtestis barrier that may impede seeding of the testicular parenchyma. Testicular involvement usually occurs contiguous to the epididymal involvement.[7]

Infertility results from either a direct obstruction by granulomatous masses in the epididymis or vas deferens or from scarring and distortion of normal anatomy. The patients may have no recollection of an acute infection and, in fact, the infection may never have had an acute manifestation. The diagnosis is suspected on finding nodules within the epididymis or nodularity in the vas deferens. This often needs confirmation with a percutaneous fine needle aspiration biopsy or excision of the epididymis. In a retrospective study of 11 cases of confirmed and treated epididymal tuberculosis, Gueye *et al.*,[8] found that the commonest manifestation was a chronic epididymal nodule and the diagnosis could only be confirmed thorough a histopathological examination of the excised epididymis. Shah described a series of 34 men with genital tuberculosis, 10 of whom had epididymal inflammation without an abscess. These men also had a normal volume of ejaculate. They were empirically treated with anti-tubercular therapy and their epididymal inflammation resolved with reappearance of sperm in the ejaculate. He concluded that epididymal inflammation due to tuberculosis could resolve using empirical antitubercular therapy and steroids in this subgroup of patients and they should not be biopsied.[9]

An ultrasonographic examination of the scrotum may reveal diffuse hypoechogenicity in epididymal and testicular inflammation. Chung *et al.*,[7] evaluated 18 patients with epididymal tuberculosis and noted that the lesions involved either a part or whole of the epididymis with enlargement and decreased echogenicity of the affected part in the majority of cases. In cases where the testis was also involved, it was either diffusely hypoechoic or had specific areas of heterogeneity.

Involvement of the vas deferens is considered one of the principal sources of genital tuberculosis. Retrograde spread of infection may occur due to bacterial invasion or reflux of urine through the ejaculatory ducts and into the vas deferens. Clinically, this manifests as multiple nodules in the palpable, scrotal part of the vas and is often bilateral. Infertility results from the direct obstruction caused by these nodules.

### Seminal vesicles/prostate/ejaculatory ducts

The seminal vesicles, prostate and the ejaculatory ducts exist in such close association that it is usually not possible to isolate the involvement of one from the other in the causation of infertility. Clinically, this involvement may manifest in one of two manners.

Inflammation and scarring may occasionally be restricted to a discrete terminal portion of the ejaculatory ducts near their opening into the prostatic urethra. Obstruction at this level results in dilatation of the proximal ductal system including the vas deferens and seminal vesicles. Seminal vesicle secretions make up the bulk of the ejaculate, contain fructose and alkalinize the ejaculate. Obstruction at the level of the ejaculatory ducts prevents seminal vesicle secretions from reaching the ejaculate. The patients thus present with azoospermia and a low-volume ejaculate or may be aspermic. Further, the ejaculate is acidic and lacks fructose. In such patients, other causes of low-volume azoospermia need to be excluded. These include congenital absence of vas deferens and retrograde ejaculation. The former can be excluded through a good clinical examination[10] while the latter can be ruled out by examining a post-ejaculatory urine sample for sperms. Absence of sperms in this sample confirms absence of retrograde ejaculation. A transrectal ultrasonogram (TRUS) is then performed to identify the site of obstruction. A discrete obstruction at the terminal part of the ejaculatory ducts is diagnosed if the proximal ductal system is dilated and aspiration of the seminal vesicles using an ultrasound guided needle reveals the presence of sperms.

Tuberculous infection of the seminal vesicles or the prostate may be diffuse and result in aspermia without a demonstrable obstruction of the ejaculatory ducts. These patients will have a clinical manifestation similar to that described for a discrete obstruction; however, the proximal ductal system will not be dilated. The disease often results in calcification of the seminal vesicles, prostate and the vas deferens. Fraietta *et al.*,[11] reported a 33-year-old man who presented with infertility 10 years after undergoing a nephrectomy for genitourinary tuberculosis. The patient reported a gradual decrease in the volume of ejaculate and was aspermic at the time of presentation. Hard prostatic nodules and enlarged seminal vesicles were palpable on rectal examination and calcifications in the prostate and seminal vesicles were confirmed on a pelvic X-ray and ultrasound examination. The ejaculatory ducts and seminal vesicles were not dilated and no fluid could be aspirated from the seminal vesicles. Absence of retrograde ejaculation was confirmed by a post-ejaculatory urine examination. The patient required testicular sperm extraction and intracytoplasmic injection for fertility.

Fibrotic, atrophic seminal vesicles with ejaculatory duct obstruction have even been considered a diagnostic feature for tuberculosis. Paick *et al.*,[12] reviewed their data on 50 men who underwent transurethral resection of the ejaculatory duct for infertility. Seventeen men had atrophic seminal vesicles on TRUS and 15 of these had a history of pulmonary tuberculosis. Vasogram was obtained on five of these men and they revealed multiple level blocks. The authors recommended that patients with atrophic seminal vesicles and a history of pulmonary tuberculosis should not be further evaluated and treated with assisted reproduction.[12] Pryor and Hendry reported similar findings in their series of 87 patients of ejaculatory duct obstruction, eight of whom were diagnosed to have tuberculosis.[13]

Rarely, non-tuberculous mycobacteria may also be responsible for causing seminal vesicle infection and infertility. Indudhara *et al.*,[14] reported a case with *Mycobacterium gastri* infection of the seminal vesicles in a diabetic patient that resulted in infertility. Semen parameters of this patient improved after antitubercular chemotherapy.

### Penile shaft

Tuberculous involvement of the penile shaft and the glans penis can result in severe disfigurement, ulcers and bulbous enlargement.[15] This may result in a deformity precluding normal sexual intercourse and subsequent infertility. Urethral strictures and urethro-cutaneous fistulae may also prevent deposition of the semen into the vagina during intercourse.

## MANAGEMENT

Management of tuberculous infertility consists of two parts. The primary aim is to treat the infection and requires antitubercular therapy. Rarely, in patients with an early diagnosis of the disease who have not developed bulky granulomas causing an obstruction, this therapy may result in restoration of fertility.

In the majority of cases, the presentation is late and antitubercular therapy does not improve the fertility status. Infertility here usually results from anatomic obstruction and therapy depends on the site and feasibility of reconstruction.

### Ejaculatory duct obstruction

While the diffuse, fibrotic type of tuberculous lesions of the ejaculatory duct and seminal vesicles are not amenable to surgery, patients with discrete obstructions at the distal end of the ejaculatory duct may benefit. The surgery in these cases involves resection of the obstructed terminal segment and marsupialization of the dilated portion of the ducts into the urethra.

The ejaculatory ducts open as paired structures on either side of the verumontanum. In normal individuals, these are not visible on a cystourethroscopic examination. During a transurethral resection of the ejaculatory ducts (TURED), the location of these openings can be identified by instilling a colored dye into the seminal vesicles through an ultrasound guided needle. This needle is used to first aspirate the seminal vesicle fluid to confirm the presence of sperms and then to instill the dye, immediately prior to the TURED. In the lithotomy position, the assistant places a finger in the patient's rectum and applies pressure over the seminal vesicles, forcing the dye to extrude through the opening. Once the opening is identified, it is resected with a cautery loop using a resectoscope. The resection is often carried deep and laterally into the prostatic tissue to ensure a wide-mouthed opening. An alternative method of dye instillation is through a vasotomy incision made for performing a vasogram. This technique, however, has a greater likelihood of causing inflammation within the vas deferens.

An alternative technique uses a combined incision-resection (TUIRED) process to avoid the instillation of dye into the seminal vesicles. During urethroscopy, delicate cuts are made with a cold knife, lateral to the verumontanum at the expected site of the ejaculatory ducts. A finger may be placed in the patient's rectum to apply pressure over the seminal vesicles to help extrude the collected seminal secretions and also help gauge the depth of the incision and prevent rectal injury. Entry into the ejaculatory ducts is confirmed when thick secretions emanate from the incision. The opening is then widened either using the cold knife or resected using the resectoscope and loop. A Foley type catheter is left in the bladder for the first postoperative day. We usually recommend examination of the semen within the first week. Presence of sperms confirms the adequacy of incision and diagnosis.

TURED or TUIRED incisions have a propensity to close over a period of time. We therefore routinely advise patients with a successful procedure to follow up regularly with semen analysis and proceed to assisted reproduction with an Intrauterine insemination (IUI), In-vitro fertilization (IVF) if the initial few attempts at spontaneous pregnancy fail.

### Epididymal obstruction

Obstruction to the epididymis may be due to the presence of nodules and masses or may be the result of a distorted anatomy. Patients with palpable masses usually require excision of the epididymis, both for diagnosis and therapy of tuberculosis. This precludes surgical reconstruction in most cases.

Patients who have obstructive azoospermia with normal volume, fructose-positive ejaculate may have discrete obstruction either within the vas deferens or at the vaso-epididymal junction. These patients should undergo surgical exploration for a possible microsurgical reconstruction.

If a nodule is palpable in the vas deferens, the vas is incised proximal and distal to the site of the nodule. Distal patency of the vas is confirmed either through the injection of saline or a formal vasogram is obtained using contrast material. Fluid from the proximal part of the vas is sampled for the presence of sperms. Presence of sperms and a patent distal vas is an indication for a vasovasal anastomosis. However, this is feasible in an extreme minority of cases as the most common lesion is a multiple site obstruction that is not amenable to reconstruction. Occasionally, it is possible to bypass multiple segmental obstructions including one at the vaso-epididymal junction by performing a vasoepididymal anastomosis between the epididymal tubule and the patent distal vas deferens.

### Assisted reproduction

The majority of patients with tuberculous infertility will require assisted reproduction. Since the most common manifestation of tuberculous infertility is azoospermia, the intervention has to be either *in-vitro* fertilization or Intracytoplasmic sperm injection (ICSI). The site of sperm harvest depends on the site of infection and the degree of destruction. Fortunately, the testis is usually spared in these cases and testicular sperm is almost always available for aspiration. Tuberculous obstruction of the genital tract does not adversely influence the outcome of assisted reproduction techniques using epididymal or testicular sperm. In a comparison of outcomes of ICSI in seven men with tuberculous obstruction versus another 37 with other indications for ICSI, the rates of fertilization and pregnancy were found to be similar.[16] Kondoh *et al.*,[17] similarly reported successful Testicular sperm extraction (TESE) and ICSI in an azoospermic man with tuberculosis, seminal vesicle calcification and epididymal nodules. This patient had failed two previous attempts at Microscopic epididymal sperm aspiration (MESA).

## CONCLUSIONS

Tuberculosis is an uncommon cause of male infertility, however, its diagnosis is important for managing not just infertility but also the systemic ramifications of the disease. In the absence of discrete nodules or granulomas, the diagnosis is based on a suggestive history and bacteriologic examination. Most cases of tuberculous infertility are not amenable to surgical correction. A rare exception is discrete ejaculatory duct obstruction that may respond to transurethral resection. Most other cases will require assisted reproduction which provides results comparable to those in men without this disease.
